# Design and manufacturability data of automatic lotus fiber extractor

**DOI:** 10.1016/j.dib.2024.110175

**Published:** 2024-02-08

**Authors:** Ngoc-Kien Nguyen, Van-Tinh Nguyen

**Affiliations:** School of Mechanical Engineering, Hanoi University of Science and Technology, Viet Nam

**Keywords:** Lotus fibre, Machine, Design, Modularization, Automation

## Abstract

Lotus silk is a type of textile and is considered as one of the most expensive fabrics in the world. It is made by weaving lotus fibres which are popularly extracted from lotus stems in some Asian countries such as Vietnam, Myanmar, Cambodia, etc. This dataset consists of the design and manufacturability data for automatic lotus fiber extraction machine including three modules (1) workpiece feeding, (2) fibre pulling, and (3) fibre spinning. The diagram of the machine principle is built in AutoCad while the 3D model was designed in Solidworks and some parts were collected from open-source library such as MISUMI, GrabCAD, and AIRTAC. The connection diagram for the HMI is designed in AutoCad and the PLC program is constructed in TIA portal. In addition, the manufacturing cost is also provided for a purpose of reference if the other researchers are interested in developing this machine. This dataset consists of (1) diagram of principle, (2) the CAD file for the design, (3) the pdf file for the connection diagram of the HMI and the PLC program, and (4) the manufacturing cost excel file. This dataset has the potential to promote the future innovations that improves the productivity of lotus fibre extraction process and working environment of the labours.

Specifications Table

**Table:** 

Subject	Mechanical Engineering
Specific subject area	Mechanical design, Manufacturing
Data format	Raw data
Type of data	Raw (CAD Files) Image Table (Excel worksheet)
Data collection	The 2D design was constructed in AutoCAD 2018. The 3D design was built using SolidWorks 2020. Some of 3D mechanical parts were manually collected through open-source databases. The connection diagram for the HMI is designed in AutoCad2018 and the PLC program is constructed in TIA portal V15. The machining cost is generated by costing tool in SolidWorks 2020. The 3D printed part is evaluated by Ultimaker Cura. The overall manufacturing cost is organized using Excel worksheet. Sources: 1. MISUMI (https://us.misumi-ec.com) 2. GrabCAD (grabcad.com) 3. AIRTAC (https://airtac.partcommunity.com/)
Data source location	Primary data sources: See Table 1
Data accessibility	Repository name: Design and manufacturability data of automatic lotus fibre extractor Data identification number: 10.17632/rfbf9wbrcp.2 Direct URL to data: https://data.mendeley.com/datasets/rfbf9wbrcp/2
Related research article	Nguyen, NK., Nguyen, VT. (2023). A Prototype of Lotus Fiber Extracting Machine. In Advances in Engineering Research and Application. ICERA 2022. Lecture Notes in Networks and Systems, vol 602. Springer, Cham. https://doi.org/10.1007/978-3-031-22200-9_7

## 1 Value of the Data

• This data can be used by the manufacturer to fabricate the lotus fiber extraction machine.
• Designers, engineers, and researchers can use these data as reference when developing the new design of fiber extracting mechanism.
• The other researchers may reuse this data as a starting point to improve the design of the automatic lotus fiber extraction machine. Many design parameters need to be optimized to enhance the efficiency of the machine.

## 2 Background

These data add the information in more details of design of the lotus fiber extracting machine to the previous paper [1] such as overall principle of operation, design parameters, control system and materials. In addition, these data additionally provide an estimated manufacturing cost to the other researchers before manufacturing decision-making.

## 3 Data Description

The data consists of four components:

(1) CAD files in .DWG file format: The computer-aided design file of machine principle have been included. This file describes the diagram of linkage that expresses the transmission system of the machine.
(2) CAD files in .part file format: The computer-aided design file of the whole machine structure has been included in. SLDASM, namely machine.SLDASM. For designs of components, a CAD file for each component has been included in. SLDPRT.
(3) Connection diagram for the HMI and ladder diagrams of the PLCs in .pdf: The connection diagram for the HMI describes the way that the HMI is connected to the other devices in the machine while the ladder diagrams express the PLC program to control the working process of the machine.
(4) Estimated manufacturing cost data in .xlsx file format: The manufacturing cost data comprises list of devices with cost ($), machine cost ($), and 3D printing cost ($) (Table 1).

**Table 1:** Summary of the design data collected and the corresponding sources.

Design ID	Sources	Attribution (License)
CSHH-SUS-MS5-20	https://us.misumi-ec.com/vona2/detail/221000551309/?HissuCode=CSHH-SUS-MS5-20	MISUMI License
Flat Washer (SPWF5)	https://us.misumi-ec.com/vona2/detail/110300252910/?HissuCode=SPWF5	MISUMI License
Split Lock Washer (SSLW5)	https://us.misumi-ec.com/vona2/detail/110300252730/?HissuCode=SSLW5	MISUMI License
HNTT5-5	https://us.misumi-ec.com/vona2/detail/110302246150/?HissuCode=HNTT5-5	MISUMI License
SCB5-10	https://us.misumi-ec.com/vona2/detail/110300239250/?HissuCode=SCB5-10	MISUMI License
HTBN400S5M-150	https://id.misumi-ec.com/vona2/detail/110302653030/?HissuCode=HTBN400S5M-150	MISUMI License
BOX-SSCB8-15	https://us.misumi-ec.com/vona2/detail/110302678140/?HissuCode=BOX-SSCB8-15	MISUMI License
SLW8	https://us.misumi-ec.com/vona2/detail/110300252730/?HissuCode=SLW8	MISUMI License
PWF8	https://us.misumi-ec.com/vona2/detail/110300252910/?HissuCode=PWF8	MISUMI License
6307ZZ	https://us.misumi-ec.com/vona2/detail/221000058301/?HissuCode=6307ZZ	MISUMI License
UCP206	https://us.misumi-ec.com/vona2/detail/221000345232/?HissuCode=UCP206	MISUMI License
CQSB25-75DC	https://us.misumi-ec.com/vona2/detail/221006499182/?HissuCode=CQSB25-75DC	MISUMI License
BSSZ1510-480	https://us.misumi-ec.com/vona2/detail/110300077910/	MISUMI License
FBF33-10-70	https://us.misumi-ec.com/vona2/detail/110300508750/?HissuCode=FBF33-10-70	MISUMI License
SB686ZZ	https://us.misumi-ec.com/vona2/detail/110302273360/?HissuCode=SB686ZZ&PNSearch=SB686ZZ	MISUMI License
HFSP5_2020_320	https://us.misumi-ec.com/vona2/detail/110302685840/	MISUMI License
SANN5-0.5	https://us.misumi-ec.com/vona2/detail/110300250540/?HissuCode=SANN5-0.5	MISUMI License
SCB4-15	https://us.misumi-ec.com/vona2/detail/110300239250/?HissuCode=SCB4-15	MISUMI License
STNTR8-0.75-2	https://us.misumi-ec.com/vona2/detail/110300250540/?HissuCode=STNTR8-0.75-2	MISUMI License
HNT1C-SUS-ML16	https://us.misumi-ec.com/vona2/detail/221000544435/?HissuCode=HNT1C-SUS-ML16	MISUMI License
AIRTAC_HFK25CL	https://airtac.partcommunity.com/3d-cad-models/hfk-series-air-gripper-airtac?info=airtac%2Fpneumatic_equipment%2Fcylinders%2Fair_gripper%2Fhfk_series.prj	AirTAC License
57HS7630A4	https://grabcad.com/library/nema-17-stepper-motor-44	GrabCAD License
SFU1610	https://grabcad.com/library/sfu1610-ballscrew-resizable-1	GrabCAD License
DSG16H	https://grabcad.com/library/dsg16h-3	GrabCAD License

## 4 Experimental Design, Materials and Methods

This dataset comprises four components that were generated as discussed next.

(1) 2D CAD data: The 2D CAD data is manually designed in AutoCad2018.
(2) 3D CAD data: The 3D CAD data was manually collected from open-source websites and online repositories. Specifically, we searched through websites such as MISUMI, GrabCAD, and AIRTAC. We used the following keywords in our search: (1) ‘motor‘, (2) ‘Spring Washers,’ (3) ‘Aluminum Extrusion,’ and (4) ‘air gripper.’ A summary of the designs gathered, and the corresponding sources and attributions are presented in Table 1. Additionally, we referenced each source throughout our research to keep up to date with the latest versions of each design.
(3) Connection and ladder diagrams for the HMI and the PLCs: The connection diagram for the HMI is manually designed in AutoCad2018. The ladder diagrams for the PLCs is built up in TIA portal V15.
(4) Manufacturing cost: The manufacturing cost of the automatic lotus fiber extraction machine is built up by the authors which is summary of the price of the used devices and the machining cost. The machining cost is determined by the costing tool of SolidWorks. The material of the machine is aluminum 6063-T5 which has the density of 2,7g/cm^3^, the machining cost for each kilogram of aluminum was assumed to be $10 and the material cost was added to obtain the total cost of the machined parts. The 3D printing cost is calculated by multiplying the printing cost for each hour and the total 3D printing time in which the printing cost is assumed to be $8 per hour for ABS material, the 3D printing time is estimated by Ultimaker Cura software V5.0 with infill density of 50 %.

## Limitations

Not applicable.

## Ethics Statement

The designs in this repository were developed by the creators listed in Table 1 and were available under the open-source licenses listed in Table 1 for non-commercial reuse. For designs retrieved from GrabCAD, consent was obtained from the creators to reuse the designs unless otherwise noted in Table 1. Any findings or opinions in this paper reflect those of the authors and not necessarily those of any designer/creator.

## CRediT authorship contribution statement

**Ngoc-Kien Nguyen:** Conceptualization, Methodology, Software, Writing – review & editing. **Van-Tinh Nguyen:** Data curation, Writing – original draft.

## Data Availability

Design and manufacturability data of automatic lotus fibre extractor (Original data) (Mendeley Data).
